# Cardiac Dysrhythmia Associated With Opioid Toxicity

**DOI:** 10.7759/cureus.8243

**Published:** 2020-05-22

**Authors:** Muhammad Shabbir Rawala, Rajat Gulati, Syed Rizvi

**Affiliations:** 1 Internal Medicine, Charleston Area Medical Center, Charleston, USA; 2 Internal Medicine, Rapides Regional Medical Center, Alexandria, USA; 3 Cardiology, Rapides Regional Medical Center, Alexandria, USA

**Keywords:** cardiac dysrhythmia, long qt, irritable bowel syndrome

## Abstract

Irritable bowel syndrome (IBS) is a functional gastrointestinal disorder involving abnormal motility. The patients are commonly started on symptom control management for IBS - diarrhea subtype by prescribing antimotility agents, usually having opioid agonist activity, and newer medications have been emerging for this as well. Patients sometimes self-medicate themselves to exceedingly high doses of these medications to achieve symptoms control. There are only a few cases of opioid-induced arrhythmia in the literature, primarily loperamide being used as a drug substitute by substance abusers. Still, it has been rarely reported to cause arrhythmia in a patient with IBS.

We present a case of a 33-year-old female with a past medical history of hypertension and depression who presented to the emergency department for evaluation of syncope. She had wide complex tachycardia on electrocardiogram (EKG) with prolonged rate-corrected QT interval (QTc). Her medications, including eluxadoline, Lomotil, and loperamide, were held and she was discharged on mexiletine with normal QTc. She did not have any more incidences of arrhythmia.

This case highlights the importance of not overdosing on opioid agonist medications prescribed to treat IBS as these can lead to potentially fatal complications. Physicians have to be judicious in promptly determining that the cause of arrhythmia can also be over-the-counter (OTC) medications.

## Introduction

The use of opioid-containing medications has been increasing globally. Most are regulated; however, a few of them are readily available over-the-counter (OTC) due to the low dose of opioids in them and without central effects. Irritable bowel syndrome (IBS) is a functional gastrointestinal disorder and is estimated to affect approximately 11% of the world’s population [1]. IBS is characterized by many subtypes, but our focus is the diarrhea-predominant type (IBS-D).

The mainstay of IBS-D treatment is lifestyle modification along with pharmacologic therapies. Amongst many of the pharmacological treatments are loperamide and Lomotil, with eluxadoline being recently approved [2].

Loperamide is peripherally acting, μ-opioid receptor agonist used for the treatment of diarrhea. Eluxadoline is a mixed mu-opioid receptor agonist with delta-opioid receptor antagonist activity and kappa-opioid agonist. Lomotil (diphenoxylate and atropine) have opioid and anticholinergic activity [2-4].

In November 2016, the US Food & Drug Administration (FDA) released a statement highlighting the potential heart effects and risk of death with high doses of loperamide [5]. The recommended dose of loperamide is usually 8 mg/day; however, in higher amounts, it can theoretically cause symptoms of opioid toxicity, including rate-corrected QT interval (QTc) prolongation. We present a case of a young female affected by IBS-D. She had been self-medicating with an exceedingly high dose of OTC loperamide, causing her to have syncope secondary to dysrhythmia.

## Case presentation

**Figure 1:**
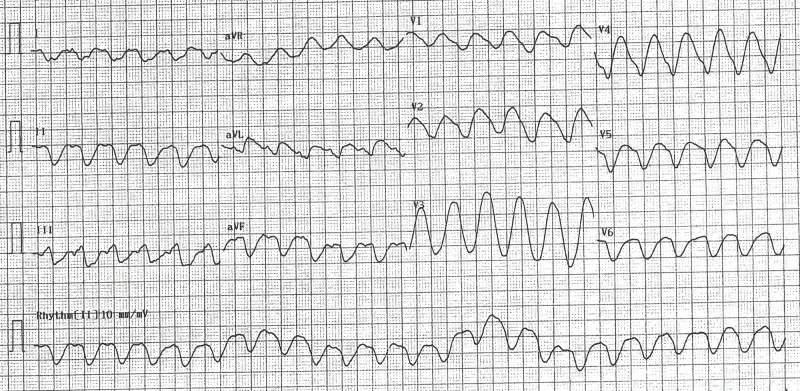
Electrocardiogram with QTc of 647 milliseconds and wide complex tachycardia

The patient is a 33-year-old female with a past medical history of hypertension, depression, and IBS who presented to the emergency department (ED) for evaluation of syncope. She was transferred from an outside facility for a higher level of care. The patient was evaluated in the ED and found to have an electrocardiogram (EKG) with QTc of 647 milliseconds and wide complex tachycardia (Figure 1). She was recently placed on eluxadoline, which has opioid agonist activity and was taking supra-therapeutic doses of loperamide and Lomotil to gain symptom control of IBS. On examination, the patient’s physical exam findings were unremarkable, including orthostatic signs. On her cardiac exam, her S1 and S2 were audible, no murmurs, no rubs or gallops were appreciated. She was admitted to the intensive care unit for observation and started on amiodarone intravenously.

**Figure 2:**
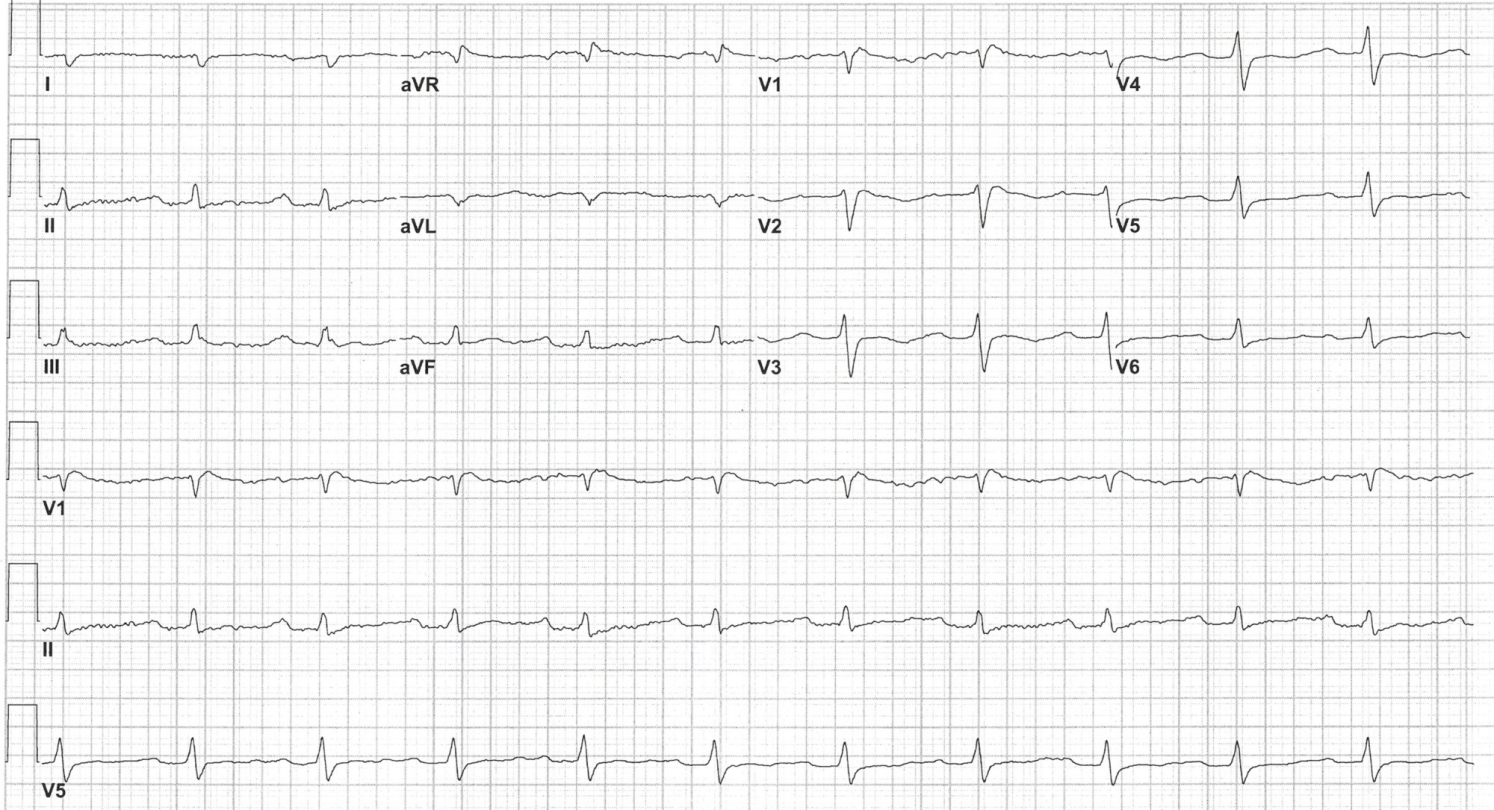
Electrocardiogram showing sinus bradycardia and first-degree atrioventricular (AV) block

The patient’s initial workup revealed normal electrolytes, no family history, or any prior history of prolonged QTc or cardiac arrhythmias. She had been taking duloxetine for her depression for a long time, which had been held at the time of admission. Her medications, including eluxadoline, loperamide, and Lomotil, were believed to be causing prolong QTc as the patient had been taking supratherapeutic doses of each with approximately 100 tablets of loperamide and 20-25 tablets of eluxadoline; therefore, these were held, and QT interval spontaneously improved in a few days. The subsequent EKGs showed sinus bradycardia and first-degree atrioventricular (AV) block (Figure 2). Echocardiogram identified normal ejection fraction and did rule out cardiomyopathy.

The patient was evaluated by an electrophysiologist who had recommended holding loperamide, Lomotil, and eluxadoline on discharge. The patient was started on mexiletine to shorten QTc interval as even after holding her home medications, QTc was borderline on higher limits. She was discharged on mexiletine with a normal QTc.

## Discussion

It was believed that potential opioid toxicity due to multiple medications having opioid agonist activity was responsible for prolonged QTc and arrhythmia.

The FDA has received formal reports of 48 cases (since 1976) of cardiovascular morbidity and mortality associated with the use of loperamide. Eventually, in June of 2016, they released a warning statement about the high-dose of loperamide and its association with a QT interval prolongation [5].

Many cases in the literature have reported ventricular arrhythmias and torsade de pointes, which resolved after discontinuing loperamide therapy [6,7]. In a small series of five patients, high dose chronic use of loperamide was shown to be associated with QRS and QTc prolongation, and a variety of events with ventricular dysrhythmias [8]. In all the case reports reviewed, the dosage of loperamide varied, ranging from 60 mg to 400 mg per day, which is far above the recommended daily dose. The maximum approved daily dose for adults is 8 mg per day for OTC use and 16 mg per day for prescription use.

The potential mechanism behind this toxicity includes inhibition of potassium channels and, therefore, QTc prolongation leading to dysrhythmia. The actual mechanism, however, is still uncertain. One study showed loperamide induced QRS and QT interval prolongation and subsequent arrhythmias from high-affinity inhibition of cardiac sodium channel (Nav1. 5) and human ether-a-go-related gene (hERG), one of the two main repolarizing cardiac K+ channels of the human heart [9]. Another study (small case series) postulated that there is a dose-dependent inhibition of inward-rectifier potassium ion channel [8]. These effects are usually seen with supratherapeutic doses of the drug [10].

Because of the lack of sufficient data in the literature, it is unclear at what doses the cardiac action potential starts getting affected by loperamide. In our case, the potential temporal association with medications and cardiac dysrhythmia possibly relates to opioid activity as the etiology of dysrhythmia, especially as QTc prolongation resolved after withholding medications.

The patient self-medicated herself on OTC medications in addition to euxadoline to achieve symptom control of her IBS. We believe better education and awareness regarding medication overdosing could have prevented this episode as it could have very quickly turned into a fatal consequence if the wide complex tachycardia had converted into a lethal arrhythmia such as ventricular fibrillation. The patient had assumed the OTC medications to be safe due to its accessibility but was unaware of the consequences of exceeding the recommended dosages.

## Conclusions

There are only a few cases of opioid-induced arrhythmia in the literature, and the presentation is still a rare occurrence. Our case highlights the importance of not combining and overdosing on opioid agonist medications being prescribed to treat IBS, as these can lead to potentially fatal complications.
